# Applications of Engineered DNA-Binding Molecules Such as TAL Proteins and the CRISPR/Cas System in Biology Research

**DOI:** 10.3390/ijms161023143

**Published:** 2015-09-24

**Authors:** Toshitsugu Fujita, Hodaka Fujii

**Affiliations:** Chromatin Biochemistry Research Group, Combined Program on Microbiology and Immunology, Research Institute for Microbial Diseases, Osaka University, Suita, Osaka 565-0871, Japan; E-Mail: tf1023jp@biken.osaka-u.ac.jp

**Keywords:** TAL, TALE, CRISPR/Cas, Cas9, dCas9, sgRNA, transcription, live imaging, locus-specific ChIP, enChIP

## Abstract

Engineered DNA-binding molecules such as transcription activator-like effector (TAL or TALE) proteins and the clustered regularly interspaced short palindromic repeats (CRISPR) and CRISPR-associated proteins (Cas) (CRISPR/Cas) system have been used extensively for genome editing in cells of various types and species. The sequence-specific DNA-binding activities of these engineered DNA-binding molecules can also be utilized for other purposes, such as transcriptional activation, transcriptional repression, chromatin modification, visualization of genomic regions, and isolation of chromatin in a locus-specific manner. In this review, we describe applications of these engineered DNA-binding molecules for biological purposes other than genome editing.

## 1. Introduction

Genome editing has been used extensively in multiple fields of biology to knock in or knock out genes of interest. The recent development of meganucleases such as *I-SceI* and engineered DNA-binding molecules such as zinc finger (ZF) proteins, transcription activator-like effector (TAL or TALE) proteins, and the clustered regularly interspaced short palindromic repeats (CRISPR) and CRISPR-associated proteins (Cas) (CRISPR/Cas) system has enabled easier genome editing in target genomic regions in cells of various types and species [1,2,3]. Such editing can be achieved either by fusion of these DNA-binding molecules to restriction endonucleases or by intrinsic nuclease activity [1,2,3]. Moreover, the applications of these engineered DNA-binding molecules have not been restricted to their use as genome editing tools. For example, by fusion with various molecules, engineered DNA-binding molecules can localize fused molecules to given loci, and fusion with transcriptional regulators and fluorescent proteins allows locus-specific artificial transcriptional control and visualization. In addition, we have applied these engineered DNA-binding molecules to a locus-tagging strategy and subsequent isolation of the tagged loci from cells.

ZF proteins are the prototypes of engineered DNA-binding molecules, but technical expertise and experience are required for optimal selection and assembly of their DNA-binding modules to generate functional DNA-binding domains. Consequently, ZF proteins have not been widely used for genome editing and other applications. By contrast, design of DNA-binding domains using TAL proteins or the CRISPR/Cas system is more straightforward, which is one reason why both molecules are in wider use today. In light of the current situation, in this review, we describe various applications of TAL proteins and the CRISPR/Cas system to biological research purposes other than genome editing.

## 2. General Information on TAL and CRISPR/Cas

The discovery and development of TAL proteins and the CRISPR/Cas system as genome editing tools have been described in detail in previous reviews [1,2,4,5,6,7,8]. Therefore, in this section, we will restrict ourselves to a brief general introduction to TAL and CRISPR/Cas.

TAL is a transcription factor of plant pathogenic bacteria such as *Xanthomonas* sp. This protein typically recognizes specific DNA sequences, about 20 bases in length, via a DNA-recognition domain consisting of assembly of DNA-binding modules corresponding to each nucleotide. Each DNA-binding module consists of a conserved repetitive region of 33–35 amino acids, of which the 12th and 13th amino acid positions (referred to as repeat variable di-residues, RVDs) are decisive in recognition of the cognate nucleotide. Thus, by combining the corresponding DNA-binding modules, one can construct TAL proteins that recognize specific DNA sequences of interest. To efficiently assemble the DNA-binding modules, the Golden Gate assembly system has been widely used [9]. In addition, high-throughput assembly systems such as fast ligation-based automatable solid-phase high-throughput (FLASH) were developed to enable rapid and cost-effective assembly of the DNA-binding modules [10]. TAL proteins linked to the endonuclease *Fok* I have been used as TALEN, a locus-specific genome editing tool.

The CRISPR/Cas system, consisting of Cas9 and guide RNA (gRNA), was discovered as a component of the bacterial adaptive immune system [6,11,12]. The Cas9/gRNA complex recognizes a DNA sequence containing the protospacer adjacent motif (PAM) sequence (e.g., 5′-NGG-3′ for CRISPR/Cas from *Streptococcus pyogenes* (*S. pyogenes*)) and the immediately upstream 20 bases complementary to the gRNA. Following DNA recognition, Cas9 cleaves the DNA sequence via its intrinsic nuclease activity. For genome editing and other purposes, the CRISPR/Cas system from *S. pyogenes* has been used most often. The original gRNA is composed of two RNA molecules: the CRISPR RNA (crRNA), possessing the sequence complementary to the target DNA sequence, and the trans-activating crRNA (tracrRNA). One can target a given genomic site by designing an RNA sequence complementary to a 20-base DNA sequence 5′-adjacent to the PAM. Recently, a single chimeric guide RNA (sgRNA) mimicking the structure of the annealed crRNA/tracrRNA has become more widely used than crRNA/tracrRNA, because the former approach entails a simplified system with only two components. sgRNA expression vectors can be constructed time- and cost-effectively using standard methods of genetic engineering. A catalytically inactive form of Cas9 (dCas9), in which two point mutations (D10A and H840A) are introduced, has been widely used for biological research purposes other than genome editing (see Section 3, Section 4, Section 5 and Section 6).

CRISPR/Cas has one major limitation, namely, that a PAM must be adjacent to the target sequences. By contrast, because TAL does not have such strict sequence limitations, the target DNA sequences can be chosen more flexibly. However, a recent report showed that the specificity of PAM recognition by CRISPR/Cas can be altered intentionally [13], potentially overcoming the aforementioned disadvantage of CRISPR/Cas and enabling target DNA sequences for CRISPR/Cas to be chosen more flexibly. In addition, the large size of the open reading frame for *S. pyogenes* Cas9 (~4.2 kbp) might be problematic to be packaged into routinely used delivery vehicles such as adeno-associated virus. In this regard, use of smaller Cas9 orthologues (e.g., Cas9 from *Staphylococcus aureus*) can overcome this disadvantage [14].

## 3. Applications of TAL and CRISPR/Cas to Locus-Specific Transcriptional Regulation

**Figure 1:**
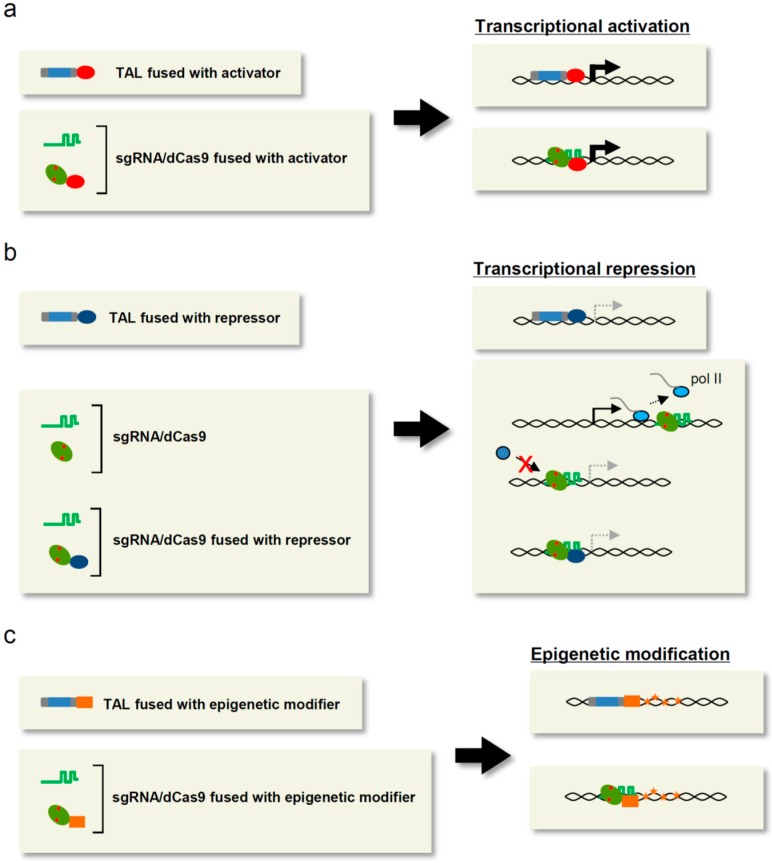
Applications of TAL and CRISPR/Cas to transcriptional/epigenetic regulation. (**a**) Locus-specific transcriptional activation system. Transcriptional activators fused with TAL or dCas9 increase the transcription of target genes. TAL: grey and blue rectangle. The blue rectangle is the DNA-binding domain; sgRNA: green line; dCas9: green ellipse with two red dots. The red dots indicate point mutations; transcriptional activator: red ellipse; activated transcription: thick bent arrow; (**b**) Locus-specific transcriptional repression system. Transcriptional repressors fused with TAL or dCas9 suppress the transcription of target genes. Alternatively, it is possible to block elongation of RNA polymerase II (pol II) or recruitment of transcriptional regulators to attenuate the transcription. Transcriptional repressor: blue ellipse; pol II: light blue circle; transcriptional regulator: dark blue circle; transcription: bent arrow; suppressed transcription: dashed bent arrow; (**c**) Locus-specific modification of epigenetic marks. Epigenetic modifiers fused with TAL or dCas9 alter epigenetic marks on target genes. These modifications may alter the transcriptional status of the relevant genes. Epigenetic modifier: orange rectangle; epigenetic modifications: yellow stars.

One of the major applications of TAL and CRISPR/Cas other than genome editing is locus-specific artificial regulation of transcription to dissect the physiological importance of gene products or manipulate biological outputs such as cellular differentiation or reprogramming. In this section, we describe various modes of artificial transcriptional regulation using TAL and CRISPR/Cas (Figure 1).

### 3.1. Transcriptional Activation

To establish an artificial transcriptional activator, a TAL protein was linked to the activation domain of VP16, a transcriptional activation protein of herpes virus [15], and used to activate the endogenous *NTF3* gene in human 293 cells [16] (Figure 1a). The resultant TAL activator (TALa), whose DNA-binding domain recognized a sequence in the *NTF3* promoter region, induced production of high levels of *NTF3* transcript and protein. Likewise, a TAL protein fused with VP64, which is composed of four tandem copies of VP16, was used as a TALa in another study [17]. In this case, the TALa induced transcription of pluripotency genes, including *SOX2*, *KLF4*, *c-MYC* and *OCT4*, by 40-fold in an exogenous reporter gene system in 293FT cells. In addition, the endogenous *SOX2* and *KLF4* transcripts could also be elevated several-fold using TALa proteins that targeted their promoters directly. Although TALa targeting the epigenetically silenced proximal promoter region did not activate *Oct4* transcription in mouse neural stem cells [18], a TALa targeting the distal enhancer ~2.4 kbp upstream from the transcription start site (TSS) could activate transcription in mouse embryonic fibroblasts (MEFs) [19]. These results demonstrate that it is feasible to use TAL proteins fused to transcriptional activators to achieve locus-specific transcriptional activation, and that genomic position and epigenetic status should be considered in the selection of target sequences.

CRISPR/Cas has also been applied to locus-specific transcriptional activation. First, Mali *et al.* [20] generated the CRISPR activator system (CRISPRa) by linking VP64 to dCas9 (dCas9-VP64) (Figure 1a). In this system, dCas9 delivers the fused protein to the sgRNA-mediated target site without cleaving the DNA. Co-transfection of 293T cells with dCas9-VP64 and an sgRNA targeting promoter regions induced strong expression of exogenous reporter genes but weak or negligible expression of endogenous *REX1*, *OCT4*, *SOX2*, and *NANOG*. By contrast, when multiple (~10) sites in these genes were targeted simultaneously, strong induction of endogenous gene transcription was detected as a result of synergistic effects on transcriptional activation. In *Escherichia coli* (*E. coli*), dCas9 fused with the ω subunit of RNA polymerase, which promotes transcription by recruiting RNA polymerase [21], was used as a CRISPRa to activate the exogenous *green fluorescent protein* (*GFP*) gene and the endogenous *lacZ* gene [22]. Thus, CRISPRa is also a feasible tool for locus-specific transcriptional activation.

### 3.2. Transcriptional Activation by Scaffold RNA System

**Figure 2:**
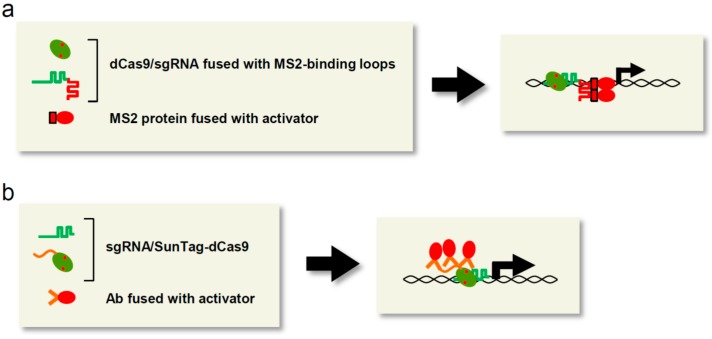
CRISPR/Cas-specific applications to transcriptional regulation. (**a**) Scaffold RNA system. Desired proteins such as transcriptional activators can be recruited to target loci via interaction with the modified sgRNA. MS2 protein: red rectangle: MS2-binding loop: red line; (**b**) The SunTag system. Desired proteins such as transcriptional activators can be recruited to target loci via interaction with the SunTag-fused dCas9. SunTag: orange line fused with dCas9; Ab: two orange straight lines.

In the CRISPR/Cas-based locus-specific transcriptional regulation system, transcriptional regulators can be tethered not only to dCas9, but also to sgRNA. For example, RNA stem loop structures that interact with the MS2 bacteriophage coat protein (MS2 or MCP) were linked to the 3′ end of sgRNA to recruit two copies of VP64-fused MS2 (MS2-VP64) [20] (Figure 2a). The resultant scaffold RNA system consisted of dCas9, the chimeric sgRNAs, and MS2-VP64. When used to target multiple sites (~10 sites) in the promoter region, but not a single site, this system could activate transcription of endogenous *REX1* as efficiently as dCas9-VP64/sgRNA.

Zalatan *et al.* [23] also utilized sgRNA as a scaffold molecule to tether VP64 to target loci. To select efficient scaffold RNA structures, they linked various structural motifs of viral RNAs, including MS2 RNA, PP7 RNA, and com RNA, which are recognized by the MCP, PP7 coat protein (PCP), and Com RNA-binding proteins, respectively, to the 3′ end of sgRNA. In both 293T cells and yeast, all combinations of dCas9 and chimeric sgRNA recruiting MCP-VP64, PCP-VP64, or Com-VP64 were functional and more effective than dCas9-VP64/sgRNA. Recently, on the basis of the observation that the tetraloop and stem loop 2 of sgRNA protrude from the Cas9/sgRNA complex, and deletion of these loops does not affect activity of CRISPR/Cas [24], Konermann *et al.* [25] developed another scaffold RNA system in which the tetraloop and stem loop 2 of sgRNA were replaced with MS2-binding RNA loops. This modified sgRNA, named sgRNA 2.0, was more than 10-fold more effective in activation of endogenous target genes than sgRNA linked to MS2-binding loops at its 3′ end. A more recent study showed that longer RNAs, such as long non-coding RNAs, can also be linked to the 3′ end of sgRNA [26].

### 3.3. Transcriptional Activation by Synergistic Regulation Systems

When TALa or CRISPRa simultaneously targets multiple (~10) sites in a gene, it can exert synergistic effects on transcriptional activation, even if each individual targeted site barely induces transcriptional activation [27,28,29,30]. Thus, gathering multiple copies of transcriptional activators on a target locus represents a better strategy for achieving the desired activation of transcription. To develop such a synergistic transcriptional activation system, dCas9 was fused with SunTag, a novel epitope tag consisting of multiple tandem repeats of the recognition sequence of the anti-GCN4 antibody (Ab) [31]. In the SunTag system, proteins fused with the single-chain variable fragment of the Ab (scFV-GCN4) are recruited to SunTag-fused dCas9 (dCas9-SunTag) (Figure 2b). The three-component system consisting of dCas9-SunTag (10 copies of the GCN4 peptide), sgRNA, and scFV-GCN4-fused VP64 induced a 10- to 50-fold increase in transcription of the endogenous *CXCR4* and *CDKN1B* genes [31]. The induced expression of CXCR4, a transmembrane protein that stimulates migration, and CDKN1B, a cell-cycle inhibitor, resulted in activation of cellular migration and suppression of cell growth, respectively, whereas dCas9-VP64/sgRNA barely affected transcription and the associated cellular phenotypes [31]. Thus, recruitment of 10 copies of VP64 to each gene by the SunTag system leads to high levels of protein expression and upregulation of physiological outputs.

In another synergistic transcriptional activation system, dCas9-VP64 was utilized together with a scaffold RNA system in which sgRNA 2.0 recruits MS2 fused to the NF-κB trans-activating subunit p65 and human heat-shock factor 1 (HSF1), which act as transcriptional activators [25]. This system, named synergistic activation mediator (SAM), robustly activated the transcription of endogenous target genes even when targeting a single site per gene. In addition, activation was much stronger than that induced by dCas9-VP64 with pools of eight sgRNAs targeting multiple sites per gene. More recently, another strategy using multiple kinds of transcriptional activators was established [32]. In this approach, dCas9 was fused to three transcriptional activators, VP64, p65 and Rta (dCas9-VPR). As expected, this system induced robust expression (87–18,000-fold increase) of endogenous genes compared to dCas9-VP64. In addition, TAL or ZF linked to VPR can be used for locus-specific activation of transcription [32].

Thus, the SunTag, SAM, and VPR systems are more effective than the single activator fusion system such as dCas9-VP64. Notably, transcriptional activation can be induced robustly only when the basal level of transcription of a target gene is low [25,32], suggesting that highly transcribed genes may already retain sufficient regulators to activate their transcription, and that such genes may not be suitable as targets for artificial transcriptional activation.

### 3.4. Transcriptional Repression

Knockout and knockdown strategies have been extensively used for loss-of-function experiments to address the physiological roles of products encoded by target genes. As mentioned briefly, TALEN and CRISPR/Cas have been widely used for knockout experiments. On the other hand, TAL or CRISPR/Cas fused to transcriptional repressors can also be used to induce loss of function (Figure 1b). To develop a TAL-mediated transcriptional repression system, TAL was fused with various transcriptional repressors and evaluated in a locus-specific transcriptional repression activity [33]. When used to target promoter regions, TAL fused with the repression domain of Krüppel-associated box (KRAB) (TAL-KRAB) exerted significant suppressive effects on transcription of endogenous *SOX2* and the miR-302/367 cluster in mammalian cells [33,34]. Double knockdown of the endogenous *c-kit* and *PU.1* genes was also achieved in mouse bone marrow cells by combinatorial use of two kinds of TAL-KRAB targeting the proximal promoter region of each gene [35]. After transplantation into irradiated recipient mice, the knockdown effects were maintained for one month in TAL-KRAB-expressing bone marrow cells.

Qi *et al.* [36] applied CRISPR/Cas as a locus-specific transcriptional repression system in *E. coli* and mammalian cells. In this approach, called CRISPR interference (CRISPRi), the dCas9/sgRNA complex is tethered to the coding region or regulatory sites bound to transcriptional regulators such as transcription factors in order to interfere with transcriptional initiation or elongation by RNA polymerase II on the target gene (Figure 1b). CRISPRi was more effective when it simultaneously targeted multiple sites proximal to the TSS than when it targeted sites distal to the TSS [36]. In contrast to its high efficiency in *E. coli* [22,36], the CRISPRi system exerted modest repressive effects (~50%) on the exogenous *GFP* gene and the endogenous miR 17–92 cluster in mammalian cells [36,37]. To improve the efficiency of CRISPRi in mammalian cells, dCas9 was fused with KRAB [38] (Figure 1b). As expected, dCas9-KRAB/sgRNA targeting of endogenous genes such as *CD71* and *CXCR4* resulted in much more effective repression (60%–80%) than conventional expression of dCas9/sgRNA in mammalian cells. CRISPRi was also feasible in *Saccharomyces cerevisiae*; in this case, dCas9 was fused to Mxi1, a mammalian transcriptional repressor domain bound to the yeast homolog of the histone deacetylase Sin3 [38]. CRISPRi has also been combined with the scaffold RNA system (see the Section 3.2) to recruit transcriptional repressors, including KRAB, to coding regions proximal to TSSs [23]. Thus, CRISPRi is useful as a novel knockdown strategy in both prokaryotic and eukaryotic cells. Because the mechanism of CRISPRi is different from that of RNA interference (RNAi), those two systems might be used as complementary methods.

Gao *et al.* [39] compared the efficiency of locus-specific transcriptional modification by TAL- and CRISPR/Cas-mediated systems targeting a single site in the *Oct4* or *Nanog* gene in MEFs. In their experimental system, TALa induced the transcription of both genes more strongly than CRISPRa when the distal enhancer region of the *Oct4* gene (~2.4 kbp) or the *Nanog* gene (~5.0 kbp) was targeted. Accordingly, active epigenetic marks at each region were more highly enriched by TALa. By contrast, the impact of transcriptional repression was comparable or higher when using CRISPRi. CRISPRi, but not TAL-KRAB, interrupted recruitment of transcriptional activators such as KLF4 and NANOG to their binding sites in the *Nanog* enhancer. Although the target sequences of the CRISPR/Cas-mediated system did not completely overlap with those of the TAL-mediated ones, due to the restriction of the PAM sequence, these results imply that when a single site is targeted with a single transcriptional effector, the TAL-mediated system is more suitable for transcriptional activation whereas the CRISPR-mediated system is more effective for transcriptional repression.

### 3.5. Epigenetic Modification

Because epigenetic marks on promoter regions or distal regulatory regions are associated with the transcriptional states of the relevant genes, artificial alteration of epigenetic marks can lead to desired alterations of gene transcription. On the basis of this concept, TAL- or CRISPR/Cas-mediated epigenetic modifiers have been developed (Figure 1c). For example, TAL was fused with LSD1, a histone demethylase that catalyzes the removal of H3K4 methylation, to achieve locus-specific epigenetic control leading to desired effects on transcription [40]. TAL-LSD1 reduced the H3K4me2 signal at target endogenous enhancers by several-fold, with no effects on non-target genes, in the human leukemia cell line K562. In addition, the H3K27ac signals at target loci were reduced, in accordance with a previous report that LSD1 interacts with other chromatin-modifying enzymes including histone deacetylase [41]. Moreover, the reduction of active epigenetic marks at enhancers led to reductions in the mRNA levels of some of the genes regulated by those enhancers. In addition, dCas9 fused with LSD1 (dCas9-LSD1) was used to locus-specifically delete the corresponding active epigenetic marks [42]. In mouse embryonic stem (ES) cells, dCas9-LSD1/sgRNA targeting the enhancer region of the *Tbox3* gene, which encodes a protein required for maintenance of pluripotency, effectively suppressed the gene expression and prevented maintenance of pluripotency via reduction of the H3K4me2 and H3K27ac marks on the enhancer regions. To develop another locus-specific histone modifier, dCas9 was fused to the catalytic histone acetyltransferase core domain of human E1A-associated protein p300 (dCas9-p300) [43]. By increasing the abundance of the H3K27ac mark, dCas9-p300 induced transcriptional activation of endogenous target genes in 293T cells. This p300 fusion strategy is also applicable to TAL or ZF proteins [43].

CpG methylation, an epigenetic mark associated with gene silencing, is maintained by DNA methyltransferases in mammalian cells [44]. By contrast, the active demethylation steps are initiated through oxidation of the 5-methylcytosine (5mC) to 5-hydroxymethylcytosine (5hmC) by TET family proteins. 5hmC is converted in a stepwise manner to completely remove the methylation mark. To evaluate the functional significance of CpG methylation in mammalians, TAL was fused with the catalytic domain of the TET1 hydroxylase (TAL-TET1) and used to induce locus-specific CpG demethylation [45]. TAL-TET1 significantly reduced CpG methylation on *KLF4*, *RHOXF2*, and *HBB* and restored expression of their gene products. In cancer biology, TAL-TET1 or TAL fused to DNA methyltransferase DNMT3A (TAL-DNMT3A) was used to manipulate expression of endogenous *CRMP4*, a suppressor of metastasis [46]. TAL-TET1 reduced the level CpG methylation on the *CRMP4* promoter in metastatic PC3 cells, thereby attenuating metastasis. Inversely, TAL-DNMT3A induced CpG methylation of the unmethylated *CRMP4* promoter in non-metastatic 22Rv1 cells, causing metastasis to occur.

Thus, locus-specific alteration of epigenetic marks is a powerful tool for investigating the physiological significance of specific epigenetic marks and achieving artificial transcriptional modification of the relevant genes. In some cases, TAL or dCas9 exerted stronger effects on transcription when fused to an epigenetic modification enzyme than when fused to a transcriptional activator/repressor such as VP16 or KRAB [42,43], suggesting that alteration of epigenetic marks may result in recruitment of a larger number of complexes, thereby enhancing the desired effect on transcription.

### 3.6. Off-Target Activity

It is reported that dCas9 binds to off-target sites [47,48,49,50]. Therefore, artificial transcriptional/epigenetic regulation systems could in principle cause off-target effects through recruitment of the fused transcriptional regulators to genomic regions other than the target loci. In this regard, in comparison to knockout by CRISPR/Cas, CRISPR/Cas-mediated knockdown systems function more locus-specifically, with negligible off-target effects [36,38]. It is reasonable to expect that off-target effects should be significantly lower in knockdown systems, because such effects would occur only when the constructed regulators bind to regulatory regions or around TSSs. Thus, CRISPR/Cas-mediated transcriptional/epigenetic regulation systems can be stringent. The same discussion should be true of TAL-mediated systems, although it is conceivable that TAL also binds to off-target sites [51,52].

### 3.7. Inducible Systems for Locus-Specific Transcriptional Regulation

Inducible protein expression or binding systems can be combined with the TAL- or CRISPR/Cas-mediated locus-specific transcriptional activation/repression system to achieve spatiotemporal modification of target transcription. For example, CRISPR/Cas-mediated locus-specific transcriptional activation/repression can be reversibly turned on and off when the expression of dCas9 and/or sgRNA is controlled by anhydrotetracycline-inducible promoters in *E. coli* [36] or galactose-inducible promoters in yeast [23].

Another method for inducible transcriptional activation using TAL proteins is an optogenetic two-hybrid system, light-inducible transcriptional effectors (LITEs) [53]. The LITEs system employs the light-sensitive cryptochrome 2 protein (CRY2) and its interacting partner CIB1; TAL fused with CRY2 interacts reversibly with CIB1 fused to an effector protein such as VP64 only in the presence of blue light. LITEs was used to achieve light-inducible and locus-specific regulation of target gene transcription in primary mouse neurons and in the brain of freely behaving mice [53]. This light-inducible system could also be used with other DNA-binding molecules such as CRISPR/Cas and ZF proteins, as well as epigenetic modification enzymes such as histone methyltransferases and histone deacetylases, as effector proteins [53,54]. Recently, another optogenetic control system was developed using the CRISPR/Cas system [55]. Those authors utilized the Magnet system, consisting of the paired photoswitchable proteins pMag and nMag [56], for optogenic reconstitution of Cas9 or dCas9. The N-terminal half of Cas9 or dCas9 fused with pMag interacts with the C-terminal half fused with nMag via heterodimerization of pMag and nMag in the presence of blue light. A Magnet system consisting of dCas9 and sgRNA targeting the coding region of the exogenous luciferase gene could interrupt transcription through CRISPRi effects in 293T cells [55]. In another two-hybrid binding approach, a chemical-induction system for TAL proteins was established based on dimerization of abscisic acid-insensitive (ABI) protein and pyrabactin resistance 1-like (PYL) protein in the presence of abscisic acid [53]. These results suggest that inducible protein expression or binding systems enable novel modes of transcriptional control for biological research.

### 3.8. Influence of Chromatin Structures of Target Sites

Robust transcriptional activation by TALa or CRISPRa can be easily achieved for exogenous reporter genes, but is difficult for some endogenous genes that are not transcribed in the cell of interest [17,20]. This is mainly because chromatin structures of non-transcribed genes are often epigenetically closed by DNA methylation or histone modification. Therefore, it is necessary to select binding sites for these engineered activators in genomic regions in which the chromatin structures are open. Consistent with this idea, TAL- or ZF-mediated transcriptional activators can substantially activate transcription of endogenous genes by targeting DNase I hypersensitive sites (DHSs), which are regions with open chromatin structures, in the gene promoters [28,57].

DNA recognition by TAL is susceptible to CpG methylation in the binding site [58,59], suggesting that recognition sites should be carefully chosen to avoid CpG methylation sites when TAL proteins are used. Alternatively, because the TAL DNA-binding module encoding NG (Asn and Gly) or N* (Asn only) at RVDs can recognize 5mC as well as thymine [58,59], it is possible to use this DNA-binding module for recognition of 5mC when a cytosine in the selected recognition sites is methylated. In fact, TALa recognizing 5mC in the CpG methylation site of the endogenous *Oct4* promoter had several-fold higher transcriptional activity than the corresponding TALa recognizing unmodified cytosine [60]. Another strategy for overcoming closed chromatin structures is to use epigenetic modifying enzymes or epigenetic drugs in combination. In this context, co-transfection of histone acetyltransferase p300 with TAL-VP64 exerted synergistic activation effects on transcription of endogenous *OCT4* in 293T cells [60]. In addition, when a de-methylation agent, 5-aza-2′-deoxycytidine, and a histone deacetylase inhibitor, valproic acid, were used in combination with TAL-VP16 in mouse neural stem cells, the levels of *Oct4* transcription were restored to a level similar to that in mouse ES cells [18].

It remains unknown whether CRISPR/Cas-mediated transcriptional activators are influenced by CpG methylation in their recognition sites, although CRISPR/Cas can cleave CpG-methylated sites [61]. Future studies should address the influence of CpG methylation on CRISPR/Cas-mediated transcriptional regulation.

### 3.9. Genome-Wide Approaches for Locus-Specific Transcriptional Regulation

CRISPR/Cas9-mediated transcriptional regulation systems have been applied to genome-wide screening for genes essential for cell growth, differentiation, malignant progression of disorders, or drug resistance [25,62]. Gilbert *et al.* [62] identified numerous genes essential for cell growth and sensitivity to cholera-diphtheria toxin in K562 cells by genome-wide screening using CRISPRi or CRISPRa with the SunTag system (Figure 2b) and sgRNAs targeting 15,977 protein-coding genes. Konermann *et al.* [25] utilized CRISPRa with the SAM system (see the Section 3.3), together with an sgRNA library for 70,290 target sites, for a genome-wide screen for genes whose products confer resistance to the anti-cancer drug PLX-4720 in human melanoma cells. Through this screening scheme, they succeeded in identifying known and novel genes, including *EGFR*, *PCDH7*, *ITGB5*, *ARHGEF1*, *BRCA3*, *GPR35*, and *TFAP2C*, responsible for PLX-4720 resistance. These results demonstrate that the CRISPR/Cas-mediated transcriptional regulation system in combination with an sgRNA library represents a powerful approach for genome-wide gain- or loss-of-function screening in various fields, including medical research.

### 3.10. Manipulation of Outputs of Biological Processes

One potential goal of TAL- or CRISPR/Cas-mediated locus-specific transcriptional regulation is manipulation of biological outputs such as metabolic fluxes, cellular differentiation, or cell fate reprogramming. In this context, CRISPRi was used to knock down the genes encoding components of the lactose regulatory pathway and thereby perturb expression of the downstream *LacZ* gene [36]. Combined use of CRISPRi and CRISPRa enabled artificial control of the flux of the biosynthetic pathway controlling production of the violet pigment violacein, which consists of five genes (*VioABEDC*) [63] in yeast [23]. CRISPRi/a targeting the *VioA*, *VioD* and *VioC* genes increased the fluxes into branched metabolic pathways and subsequent production of the corresponding compounds (deoxyviolacein, prodeoxyviolacein, and proviolacein).

On the other hand, TAL- or CRISPR/Cas-mediated locus-specific transcriptional regulation has been used to activate transcription of pluripotency genes including *SOX2*, *KLF4*, *c-MYC*, and *OCT4* [17,18,19,20,39,60], implying that the pluripotency genes are attractive targets for artificial transcriptional activation aimed at reprogramming cell fate in induced pluripotent stem cells (iPSCs) [64,65]. Knockdown of *OCT4* by CRISPRi led to morphological changes in human ES cells [66], suggesting that it is possible to control cellular identity by altering the pluripotency network. Exogenous expression of a single transcription factor, either neurogenin 2 (NEUROG2) or neurogenic differentiation factor 1 (NEUROD1), promotes differentiation of human iPSCs into induced neuronal cells (iNs) [67,68]. When a pool of 30 sgRNAs designed against the *NEUROG2* or *NEUROD1* gene were used together, CRISPRa was able to induce differentiation of iPSCs into iNs [32], demonstrating its utility for artificial reprogramming of cell fate decisions.

## 4. Locus Visualization for Live Imaging

**Figure 3:**
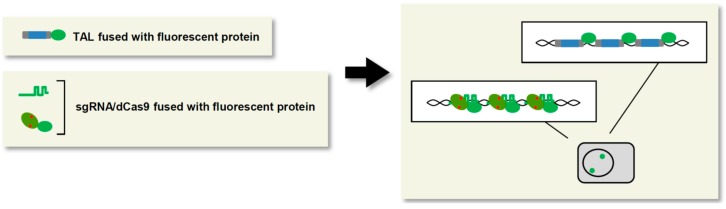
Locus visualization for live imaging. Fusion of fluorescent proteins to TAL or dCas9 enables visualization of target loci in living cells. Fluorescent protein: green ellipse; visualized loci: green dots.

Visualization of specific genomic regions is required for observation of spatiotemporal dynamics of chromosomal organization and segregation *in vivo*. To this end, fluorescence *in situ* hybridization (FISH) has been employed as a locus imaging technology [69]. Although FISH can visualize specific genomic loci, it cannot be used for live imaging because the procedure requires fixation of cells. An alternative locus visualization technology involves locus-tagging using an exogenous DNA-binding protein and its cognate DNA sequence [70,71,72]; an exogenous DNA-binding protein fused to a fluorescent protein such as GFP can label a locus of interest where its binding DNA element has been artificially inserted. This system is therefore ideal for live imaging of the chromosomal dynamics of loci. However, insertion of DNA elements by gene targeting is time-consuming. To overcome this disadvantage, locus-specific visualization systems have been developed using engineered DNA-binding molecules. In the first such study, a ZF protein was fused to GFP (ZF-GFP) [73]. By targeting the centromeric and major satellite repetitive sequences, ZF-GFP could be used to visualize the centromeres in living roots of *Arabidopsis* and major satellite regions in NIH3T3 cells, respectively. Next, a locus-specific visualization technology using TAL proteins was developed, named TAL-mediated genome visualization (TGV) [74] (Figure 3). In living mouse ES cells, TGV targeting repetitive genomic regions such as pericentromeric major satellite repeats, centromeres, and telomeres formed bright puncta co-localized with their regional marker proteins. In recognition of DNA sequences, TAL is sensitive to differences of a few nucleotides [75]. Taking advantage of this property, TGV was successfully used for allele-specific labeling of a locus in which single-nucleotide polymorphisms existed between each allele [74].

More recently, a CRISPR/Cas-based visualization system was developed [76] (Figure 3). In this system, dCas9 fused with GFP (dCas9-GFP) was used for live imaging of repetitive genomic regions such as telomeres. The vast majority (95%) of the detected fluorescent puncta were co-localized with telomeres, demonstrating the high specificity of this system. For visualization of non-repetitive genomic regions, it is necessary to simultaneously target multiple sites within a locus to allow integration of multiple copies of GFP, because the fluorescence from a single molecule of GFP is not sufficient to be observed by microscopy. One such non-repetitive genomic locus was successfully visualized by dCas9-GFP with more than 16 sgRNAs that targeted a region spanning ~5 kb within the locus [76]. In addition, the SunTag system of CRISPR/Cas (Figure 2b) has been used for locus visualization [31]. Because 24 copies of the GCN4 peptide can be linked in tandem in the SunTag [31], the SunTag system can theoretically gather 24 molecules of scFV-GCN4-fused GFP to a target locus. In fact, in comparison to the visualization system using dCas9-GFP, the SunTag system targeting telomere regions yielded brighter fluorescent puncta [31].

Thus, TAL- or CRISPR/Cas-based visualization systems are ideal tools for locus-specific live imaging. However, simultaneous targeting of multiple sites in a coding region for the purpose of locus visualization may interfere with transcription via CRISPRi effects [36]. In such cases, the observed dynamics of the locus may not precisely reflect its physiological behavior. To avoid such undesirable effects, target positions should be designed at regions far downstream or upstream from the TSS, and expression of the transcript from the targeted locus should be confirmed in the presence of the locus-tagging molecules.

## 5. Biochemical Analysis of RNAs

**Figure 4:**
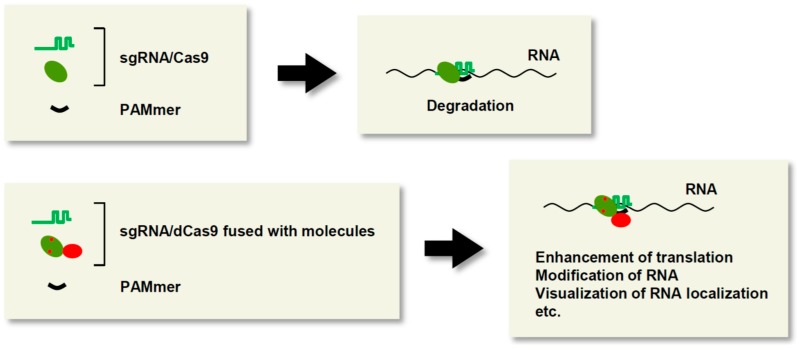
CRISPR/Cas-mediated analysis of RNA. The CRISPR/Cas system can recognize RNAs through the designed PAMmer. This system, named RCas9, can be used for various applications such as cleavage, isolation, and visualization of endogenous RNAs. Cas9: green ellipse; effector molecule (translational activator, RNA modification enzyme, fluorescent protein, *etc.*): red ellipse.

CRISPR/Cas recognizes as its substrate double-stranded DNA (dsDNA) containing the PAM sequence. Recently, O’Connell *et al.* [77] showed that CRISPR/Cas can bind with high affinity to single-stranded RNA (ssRNA) *in vitro* when the PAM is presented in *trans* as a separate DNA oligonucleotide (Figure 4). In this RNA-recognition system, the DNA oligonucleotide (referred to as a PAMmer) anneals with the PAM position of a target ssRNA and stimulates site-specific recognition of the ssRNA by the CRISPR/Cas complex. However, when both a target ssRNA and dsDNA that possesses the sequence corresponding to the ssRNA are present in a reaction, CRISPR/Cas also recognizes the dsDNA as a substrate regardless of the presence of the PAMmer. To avoid such an undesirable reaction with dsDNA, O’Connell *et al.* [77] improved the design of the PAMmer such that it annealed specifically with non-PAM sites in ssRNA, thereby maintaining the mimicked PAM structure. As a result, CRISPR/Cas specifically recognized and cleaved the 20-base 5′-adjacent to the non-PAM site in the ssRNA, but not the corresponding dsDNA sequence lacking the PAM. In addition to cleavage of ssRNA, they succeeded in isolating the endogenous *GAPDH* mRNA from HeLa cell lysate by affinity purification using biotin-tagged dCas9, sgRNA, and the improved PAMmer.

Therefore, this RNA-targeting system, named RCas9, would be useful for manipulating or analyzing RNAs for various purposes, including cleavage, isolation, modification, and imaging (Figure 4). For example, live imaging of the behavior of endogenous RNAs would enable analysis of their spatiotemporal dynamics and physiological functions. On the other hand, Csm proteins of the type III-B CRISPR/Cas system can recognize and cleave ssRNA [78,79,80], suggesting the potential utility of the type III-B system in an approach like RCas9. Nevertheless, the type III-B system requires six protein components, which may be problematic in applications aimed at RNA analysis.

## 6. Isolation of Genomic Regions of Interest for Analysis of Genome Functions

Last, we describe applications of TAL proteins or the CRISPR/Cas system to locus-tagging strategies and subsequent isolation of the tagged loci from cells. Genome functions such as transcription are regulated spatiotemporally in a locus-specific manner. To understand the molecular mechanisms of such genome functions, it would be ideal to comprehensively identify the molecules (proteins, RNA, DNA *etc.*) that interact with genomic regions of interest *in vivo*. To this end, we developed two locus-specific chromatin immunoprecipitation (locus-specific ChIP) technologies, insertional ChIP (iChIP) [81,82,83,84,85,86] and engineered DNA-binding molecule-mediated ChIP (enChIP) [87,88,89,90,91]. Locus-specific ChIP is a biochemical method for isolating target genomic regions from cells. Identification of the molecules that interact with the isolated genomic regions facilitates understanding of the molecular mechanisms underlying their functions of these loci.

**Figure 5:**
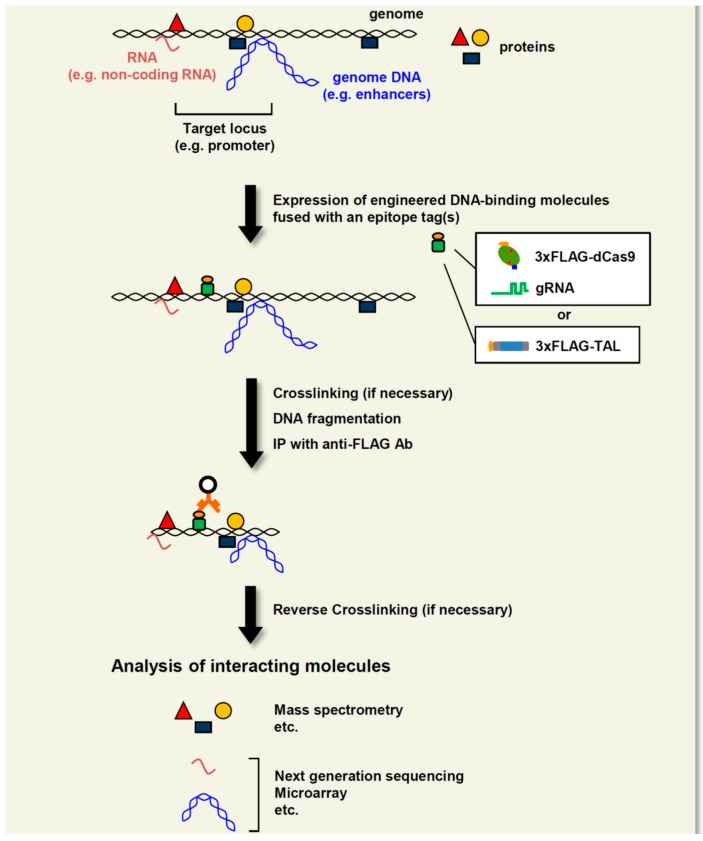
enChIP using TAL or CRISPR/Cas. To isolate genomic regions of interest from cells, TAL or dCas9/sgRNA is designed and expressed in the cells to be analyzed. The cells are crosslinked (if necessary) and lysed, and the genomic DNA is fragmented by sonication or other methods. Chromatin complexes of the target region are immunoprecipitated with an Ab against the epitope tag (e.g., 3xFLAG-tag) fused to TAL or dCas9. Alternatively, chromatin complexes of the target region can be affinity-purified with an Ab against TAL or dCas9. Comprehensive identification of molecules (DNA, RNA, proteins, *etc.*) bound to the isolated genome complexes enables elucidation of the molecular mechanisms underlying genome functions. Interacting RNA: red line; interacting genomic DNA: blue line; interacting proteins: red triangle, orange circle, and dark blue rectangle; Ab fused with a carrier: orange line fused with circle.

Locus-specific ChIP consists of a locus-tagging strategy and subsequent affinity purification of the tagged loci. In practice, exogenous DNA-binding molecules such as the bacterial DNA-binding protein LexA and its binding DNA elements are utilized for locus-tagging in iChIP [81,82,83,84,85,86], whereas recognition of endogenous DNA sequences by engineered DNA-binding molecules such as TAL and CRISPR/Cas is used for enChIP [87,88,89,90,91]. One advantage of enChIP over iChIP is that it does not require the insertion of exogenous DNA sequences for locus-tagging by gene targeting. The procedures for enChIP using TAL or CRISPR/Cas are as follows (Figure 5): (I) design of the engineered DNA-binding molecules: a TAL or dCas9/sgRNA is generated to recognize a unique DNA sequence in a genomic region of interest; (II) Locus-tagging: the designed molecule can be fused with an epitope tag(s) such as 3xFLAG-tag (3xFLAG-TAL or 3xFLAG-dCas9) and a nuclear localization signal, and then expressed in the cell to be analyzed; (III) DNA fragmentation: the resultant cell is crosslinked (if necessary) with formaldehyde or other crosslinkers and lysed, and the DNA is fragmented by sonication or other methods; (IV) Affinity purification: chromatin complexes bound to the expressed engineered DNA-binding molecule are isolated by affinity purification. Following reversal of crosslinking (if any), subsequent purification of DNA, RNA or proteins allows their identification and characterization by next-generation sequencing, microarray, or mass spectrometry.

We used enChIP using CRISPR/Cas to directly identify proteins that interact with the promoter region of *interferon regulatory factor 1* (*IRF-1*), a gene whose expression is induced by interferon γ (IFNγ) stimulation [87,89]. By combining stable isotope labeling using amino acids in cell culture (SILAC), a quantitative form of mass spectrometry, with enChIP (enChIP-SILAC), we were able to identify dozens of proteins potentially recruited to the promoter region after IFNγ stimulation in human cell lines. Several components of histone deacetylase (HDAC) complexes (e.g., RBBP4, PA2G4, and TBL3) were included as the candidate proteins, consistent with the importance of HDAC for expression of IFNγ-inducible genes [92,93]. We also succeeded in identifying proteins that interact with telomere regions in a mouse hematopoietic cell line by enChIP using TAL proteins [88]; in that study, enChIP combined with MS (enChIP-MS) enabled identification of known and novel proteins interacting with the telomere regions. KDM5X, POLA1, CTBP1, DDX54, GNL3L and BEND3, which we identified as telomere-interacting proteins, were confirmed to co-localize with telomeres [88], demonstrating the utility of this technology. In addition, we could identify telomere-interacting RNAs by combining enChIP with RT-PCR (enChIP-RT-PCR) or RNA-sequencing (enChIP-RNA-seq) [88,90]. Using these approaches, non-coding RNAs such as snoRNAs and the long non-coding RNA *Neat1* were identified as novel telomere-binding RNAs. RNA-FISH confirmed that these identified RNAs were partially co-localized with telomere regions [90]. Thus, our results demonstrated the utility of enChIP as a non-biased research tool for identifying proteins and RNAs bound to genomic regions of interest *in vivo*. In principle, it should be possible to use enChIP to identify interacting chromosomal regions, as well as proteins and RNAs (Figure 5). We are now undertaking a non-biased search for identifications of genomic regions by combining enChIP with next-generation sequencing (enChIP-seq).

In enChIP experiments, it is necessary to put appropriate controls to cancel out contamination of off-target sites. To this end, we suggest following guidelines: (1) Compare different conditions for an engineered DNA-binding molecule (e.g., presence or absence of extracellular stimulations, use of different cell types); (2) Compare several different engineered DNA-binding molecules for each target genomic region (e.g., use of different gRNAs). These controls may also be useful for aforementioned applications such as artificial transcriptional/epigenetic regulation, live imaging, and RNA analysis.

Taken together, the findings to date demonstrate that enChIP using TAL or CRISPR/Cas is a feasible tool for identifying genome-interacting molecules in a locus-specific manner and dissecting the molecular mechanisms underlying genome functions. In the near future, such approaches should make it possible to achieve genome-wide mapping of genome-interacting molecules.

## 7. Conclusions

In this review, we described various applications of engineered DNA-binding molecules such as TAL and CRISPR/Cas other than genome editing, based either on the fusion of these proteins with restriction endonucleases or their intrinsic nuclease activity. Locus-specific transcriptional regulation enables elucidation of the physiological relevance of products transcribed from the targeted genes, as well as manipulation of biological outputs such as cellular differentiation or reprogramming. Locus-specific visualization and locus-specific ChIP technologies enable dissection of spatiotemporal dynamics of chromosomal organization and/or molecular mechanisms of genome functions. RNA-specific analysis may be useful for dissection or manipulation of the physiological functions of RNAs. One advantage of CRISPR/Cas over other systems is the easier design and construction of sgRNAs. Therefore, although applications of TAL proteins preceded those of CRISPR/Cas, CRISPR/Cas can be expected to emerge as the predominant technology for future applications.
